# Reduction in cardiovascular mortality following severe hypoglycemia in individuals with type 2 diabetes: the role of a pragmatic and structured intervention: Structured intervention for community hypoglycemia

**DOI:** 10.1186/s12933-020-01204-3

**Published:** 2021-01-12

**Authors:** Sam M. Pearson, Beverley Whittam, Kavita Kulavarasalingam, Amelia Mitchell-Gears, Cathyrn James, Ramzi A. Ajjan

**Affiliations:** 1 Leeds Teaching Hospitals NHS Trust, Leeds, UK; 2 Leeds Institute of Cardiovascular and Diabetes Research, University of Leeds, Leeds, UK; 3 Calderdale and Huddersfield NHS Foundation Trust, Huddersfield, UK; 4 Yorkshire Ambulance Services, Wakefield, UK; 5 The LIGHT Laboratories, Centre for Diabetes and Vascular Research, Leeds, UK

**Keywords:** Community hypoglycemia, Structured intervention, Cardiovascular mortality

## Abstract

**Background:**

Mortality in individuals with diabetes with severe hypoglycemia requiring ambulance services intervention is high and it is unclear whether this is modifiable. Our aim was to characterise this high-risk group and assess the impact of nurse-led intervention on mortality.

**Methods:**

In this single centre study, patients with diabetes and hypoglycemia requiring ambulance call out were randomized to nurse led support (intensive arm) or managed using existing pathways (standard arm). A third group agreed to have their data collected longitudinally (observational arm). The primary outcome was all-cause mortality comparing intensive with combined standard and observational arms as well as standard arm alone.

**Results:**

Of 828 individuals identified, 323 agreed to participate with 132 assigned to intensive, 130 to standard and 61 to observational arms. Mean follow up period was 42.6 ± 15.6 months. Mortality in type 1 diabetes (n = 158) was similar across study arms but in type 2 diabetes (n = 160) this was reduced to 33% in the intensive arm compared with 51% in the combined arm (p = 0.025) and 50% in the standard arm (p = 0.06). Cardiovascular deaths, the leading cause of mortality, was lower in the intensive arm compared with combined and standard study arms (p < 0.01).

**Conclusions:**

Medium-term mortality following severe hypoglycemia requiring the assistance of emergency services is high in those with type 2 diabetes. In individuals with type 2 diabetes, nurse-led individualized intervention reduces cardiovascular mortality compared with standard care. Large-scale multicentre studies are warranted to further investigate this approach.

*Trial registration* The trial was retrospectively registered on http://www.clinicaltrials.gov with reference NCT04422145

## Background

There are two main forms of diabetes mellitus, type 1, whereby autoimmune destruction of islet β-cells renders the patient insulin deficient and type 2, largely mediated by insulin resistance and later relative insulin deficiency [1, 2].

Lowering glucose levels has been shown to reduce microvascular complications and long-term macrovascular disease in individuals with diabetes [3–8]. However, intensive glycemic control increases the risk of hypoglycemia, which is associated with adverse clinical outcome [9–12]. The ACCORD trial demonstrated excessive mortality in the intensive glucose arm of the study, necessitating study termination and fuelling speculation about the role of tight glucose control in the management of type 2 diabetes [13]. While subsequent analysis failed to conclusively demonstrate that low glucose levels caused the higher mortality in intensively-treated patients, hypoglycemia was generally associated with increased mortality in the study population [14]. There are mechanistic pathways that link hypoglycemia to increased mortality, including cardiac arrhythmias in the short term and an inflammatory-thrombotic response that predisposes to vascular events in the long-term [15–17]. In particular, a study in type 2 diabetes subjects has shown that low blood glucose levels, under controlled conditions, increases thrombosis risk for up to 1 week, providing one explanation for the relationship between cardiovascular death and hypoglycemia [16]. Indeed, large scale studies and anecdotal reports have shown associations between antecedent hypoglycemia and vascular mortality [12, 18−21].

In large population studies, the association between HbA1c and mortality has been repeatedly shown to take a U-shaped curve, raising the possibility that aggressive glycemic control increases mortality through precipitation of hypoglycemia [22, 23]. On the other hand, individuals with high HbA1c are not necessarily protected from severe hypoglycemia [24], and therefore HbA1c levels are not the sole predictors of predisposition to low glucose levels.

Our previous work has shown that individuals with severe hypoglycemia requiring ambulance services intervention are at high risk of mortality within the 1st year of the event [25]. However, the main causes of death in these individuals were unclear and the longer-term mortality, beyond 1 year, is yet to be established. More importantly, it is unknown whether the high mortality in this population is modifiable and studies in this area have not been conducted to date.

We hypothesise that a pragmatic nurse-led intervention reduces mortality in individuals with diabetes requiring ambulance services intervention. Therefore, we conducted a single centre pilot study with the following objectives:

1. Characterise in detail the cohort of patients having severe hypoglycemia requiring emergency services intervention, study mortality over a long follow up period and ascertain the cause of death.
2. Study the effects of structured nurse-led intervention, including patient education, on mortality in this population.

## Methods

### Study participants

Study participants were individuals with diabetes who experienced severe hypoglycemia requiring ambulance services call-out in the area in and around the city of Leeds, UK. For this work, “severe hypoglycemia” was defined as low glucose levels requiring assistance of emergency services and not only the help of another person as the term is commonly used. Inclusion criteria included the presence of any type of diabetes together with a recent ambulance call out for severe hypoglycemia. The only exclusion criterion was individuals without diabetes.

### Enrolment on clinical trials database

The study is enrolled on www.clinicaltrials.gov with reference number NCT04422145.

### Study procedure

Participants were asked to participate in the interventional study but given the option for enrolment in an observational study. Therefore, the study comprised interventional and observational parts with the first divided further into intensive and standard arms as follows:

- Interventional study: participants were randomized to either:
  - Intensive arm: participants received intensive nurse-led intervention for a period of 3 months with access to the nurse for a total of 12 months.
  - Standard arm: participants were managed according to local guidelines.
- Observational study: participants did not want to be randomized to receive the intervention but were willing to have their records reviewed electronically. They were returned to their standard diabetes care provider at baseline and not followed up in person by the diabetes research nurse.

Participants in the standard and observational arms of the trial therefore received the same medical care for their diabetes.

**Fig. 1:**
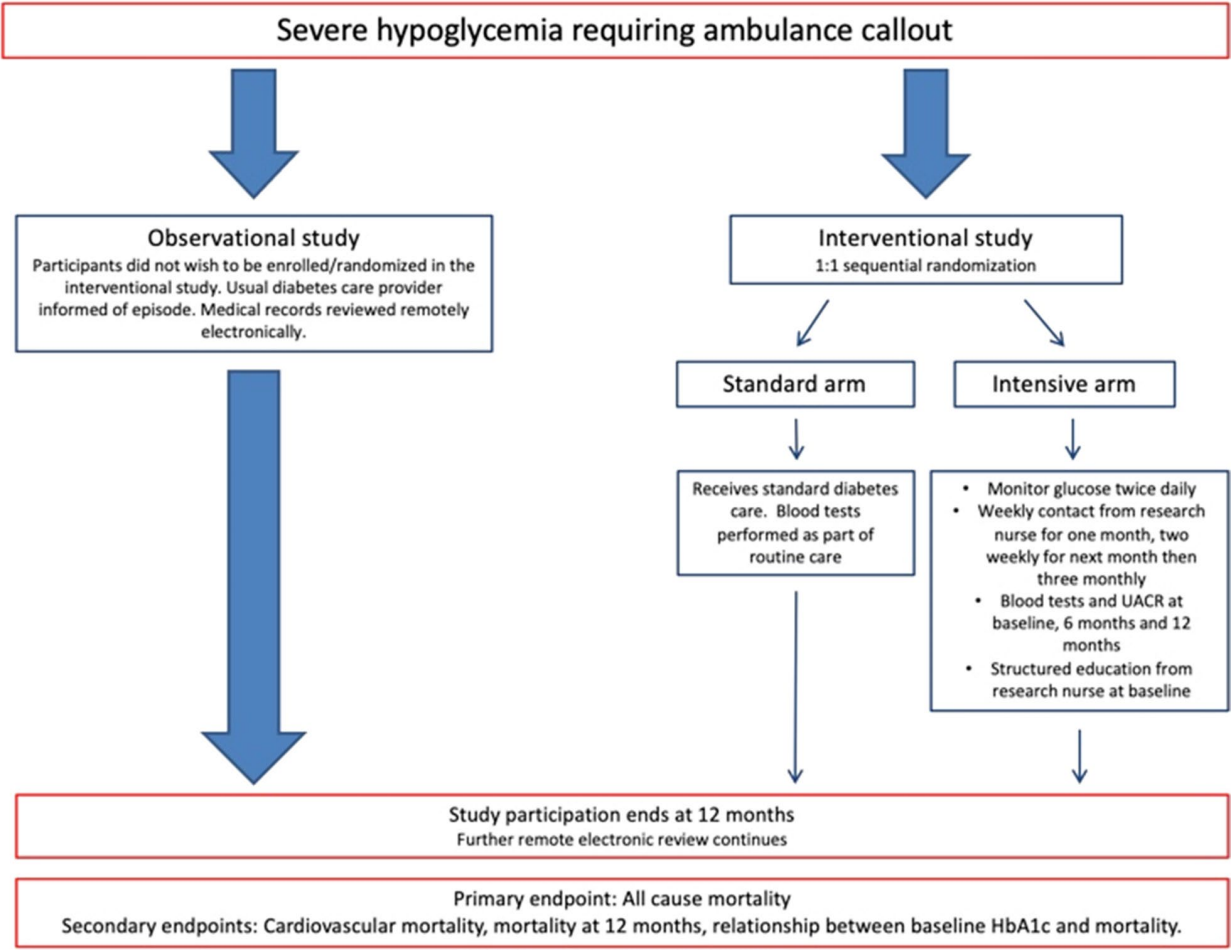
A diagrammatic depiction of trial design. Out of 828 diabetes patients suffering severe hypoglycemia, 323 agreed to participate in the study. A total of 61 participants did not wish to be involved in an interventional study and were entered into an observational arm. The remaining 262 participants were randomized to structured nurse led intervention or standard care

Routine blood tests were conducted in participants at baseline, unless blood tests were available within 3 months prior to enrolment. Blood tests were also taken at 6 and 12 months in the intensive arm whilst in those in the standard arm or the observational study, the electronic records were accessed for routine blood test results. Participants were subdivided into type 1 and 2 diabetes based on data from patient electronic records. Figure 1 displays trial design.

### Randomization and blinding

Participants in the interventional trial were sequentially randomized to receive either intensive nurse led intervention or standard care based on the date they were referred to the research team. Given the fact that the research nurse was providing education to participants, they could not be blinded to study group. The primary investigator (RA) was asked for advice only when patterns of glycemia were deemed to be clinically serious, including sustained hypoglycemia, nocturnal hypoglycemia and consistently high glucose levels (> 240 mg/dL), consistent with local practice. Investigators aware of participants allocation to study arms were not involved in data analysis.

### Role of the research nurse and structured education provided

Following recruitment to the study and randomization to the intensive education group, participants received the following structured interventions from a senior diabetes research nurse:

1. An attempt was made to ascertain the likely cause of the index episode of hypoglycemia.
2. Written and verbal information was provided regarding common causes of hypoglycemia including exercise, alcohol consumption and anti-diabetic therapies.
3. Participants using insulin were examined for lipohypertrophy and advised on the importance of avoiding injection into these sites.
4. Participants were provided with suitable equipment to measure their capillary blood glucose if they were not already doing so. They were encouraged to measure glucose at least twice daily and record results for later review.
5. A dedicated phone number was provided along with an email address for participants to contact the diabetes research team with any clinical queries they may have.

Following baseline assessment, participants were contacted weekly for the 1st month after enrolment, every 2 weeks for the 2nd month and once in the 3rd month. Two further contacts occurred at 6 and 12 months and therefore main study intervention occurred in the first 3 months of enrolment. Participants could also contact the research team on an ad-hoc basis. During the contacts outlined above, the research nurse provided the following interventions:

1. Review of capillary blood glucose results and adjustment of medications and insulin doses as necessary. Seeking advice from a diabetologist (RA) only occurred in difficult cases or if a prescription of a new hypoglycemic agent was deemed necessary (as per local guidelines). Therefore, most management decisions were made by the study nurse.
2. Further education as required regarding any of the topics discussed at the baseline assessment.

### Follow up period

All study participants were followed up for a minimum period of 12 months. When the study terminated, all participants again had their electronic records reviewed for mortality. If they were deceased, their cause of death was determined by reviewing death certificate of the participant, which are issued for all deceased individuals in the UK. In UK death certificate, the clinical condition directly related to mortality is recorded as the main cause of death (which was collected in this study) and contributing conditions are also highlighted. Cause of death is determined by a senior hospital doctor, or primary care physician, who is familiar with the patient. Data from death certificates are used on a population basis to determine national health policies.

### Endpoints

The primary endpoint was death from any cause at study end comparing the intensive study arm with combined standard and observational arms as well as the standard arm alone.

Secondary and exploratory endpoints included:

- Analyse mortality at 12 months in the whole population and separately for type 1 and type 2 diabetes individuals.
- Establish the cause of death and study differences between study arms.
- Analyse the relationship between baseline HbA1c and mortality.

### Statistical analysis and power calculations

Given our previous study [25], our initial power calculations were based on a follow up of 1 year only, which would have required 200 individuals in each group to show a drop in mortality from 18–10% secondary to the intervention (power of 80%, at p < 0.05). However, given the slower than anticipated recruitment rate, the study was subsequently revised to extend the follow-up period. Assuming a mortality rate of 30% during the extended follow up in individuals managed using existing hypoglycemic pathways, 106 individuals in each study arm were required to demonstrate a reduction in mortality to 15% in the intensive arm of the study with a power of 80% (at p < 0.05). Assuming a drop-out rate of 20%, we aimed to recruit a minimum of 130 individuals in each of the intensive and standard study arms.

Minitab v.19 (Mintab, inc, USA) was used for statistical analysis. Kaplan–Meier survival curves were made using Prism v8.3.1 (Graphpad, USA). Chi-squared test was used for testing proportions, while T-test was employed to analyse normally distributed continuous variables between two groups. When numbers of participants with a particular outcome for a categorical variable was small (≤ 5 in any category), Fishers exact test was used. For testing differences in baseline characteristics between the three groups, one-way ANOVA was used for continuous variables and Chi-squared for categorical variables. For all analysis, an intention to treat methodology was used with all participants being analysed according to their original group, independent of drop out.

Given that individuals in the standard arm and the observational study received the same care, namely their diabetes was managed by their standard healthcare provider following the episode of hypoglycemia, data was analysed in two ways:

1. Comparison between the intensive arm and the combined standard arm/observational study (termed combined group).
2. Comparison of the intensive and standard arms of the interventional study.

## Results

### Recruitment rate

A total of 828 referals to the study team were made by ambulance services, of which 98 involved referrals of the same participant more than once. After discounting these 732 prospective particpants were left. Of these, 232 of declined involvement at an initial screening phone call, 84 could not be contacted, 55 could not provide written informed consent, 48 did not attend a planned meeting with the research team, 3 died prior to contact and 1 was not contacted in error by the research team. This left 323 participants of whom 61 agreed to be followed up remotely but did not wish to be randomized to the interventional trial (observational group) and 262 participants whom were randomized to standard care or intensive intervention.

The first participant was recruited to the trial in February 2013 and the last participant was recruited in December 2017. The study was terminated after reaching the required numbers in the interventional arm together with a minimum follow up of 12 months (December 2018).

### Baseline characteristics

A total of 262 participants agreed to participate in the interventional study with an additional 61 participants participating in the observational study. Across all groups, 158 participants had type 1 diabetes (48.9%), 160 participants had type 2 diabetes (49.5%) and 5 participants (1.5%) had other forms of diabetes.

A summary of baseline characteristics is shown in Table 1.

**Table 1:** A summary of baseline patient characteristics

		Intensive (n = 132)	Standard (n = 130)	Observational (n = 61)	Difference between groups
Ethnicity (%)	White	116 (87.9)	107 (82.3)	58 (95.1)	
	Afro-Caribbean	5 (3.8)	11 (8.5)	0	
	South Asian	9 (6.8)	9 (6.9)	1 (1.6)	
	Other	2 (1.5)	3 (2.3)	2 (3.3)	
Form of diabetes (%)	Type 1 diabetes	69 (52.3)	65 (50)	24 (39.3)	p = 0.24
	Type 2 diabetes	60 (45.4)	64 (49.2)	36 (59)	p = 0.22
	Other diabetes	3 (2.3)	1 (0.8)	1 (1.6)	NA
Male gender (%)	Total	83 (62)	76 (58.5)	35 (57.4)	p = 0.68
	T1D	48 (69.6)	38 (58.5)	16 (66.7)	p = 0.40
	T2D	33 (55)	37 (57.8)	18 (50)	p = 0.75
Age (years ± SD)	Total	61.4 ± 1 8.5	62.0 ± 18.7	70.3 ± 14.0	*p = 0.03*
	T1D	50.5 ± 16.7	49.7 ± 16.5	59.7 ± 12.9	*p = 0.03*
	T2D	74.2 ± 10.7	74.84 ± 10.2	77.3 ± 9.8	p = 0.38
Presenting capillary glucose (mmol/L ± SD)	Total	2.1 ± 0.7	2.2 ± 1.0	2.1 ± 0.6	p = 0.79
	T1D	2.0 ± 0.7	2.1 ± 1.2	1.9 ± 0.6	p = 0.73
	T2D	2.3 ± 0.7	2.3 ± 0.8	2.3 ± 0.6	p = 0.94
HbA1c (mmol/mol ± SD)	Total	62.8 ± 17.1	63.5 ± 15.6	60.8 ± 17.0	p = 0.62
	T1D	66.3 ± 19.4	66.8 ± 14.5	61.3 ± 10.5	p = 0.40
	T2D	58.5 ± 13.4	60.0 ± 16.2	61.2 ± 20.4	p = 0.76
Current smoker (%)	Total	29 (22.0)	27 (20.8)	10 (16.4)	p = 0.66
	T1D	22 (31.9)	18 (27.7)	5 (20.8)	p = 0.45
	T2D	7 (11.7)	9 (14.0)	5 (13.9)	p = 0.91
Duration of diabetes (years ± SD)	Total	23.7 ± 14.0	23.1 ± 12.7	25.7 ± 13.7	p = 0.58
	T1D	26.3 ± 15.6	27.2 ± 13.1	32.0 ± 12.0	p = 0.25
	T2D	21.0 ± 11.3	19.3 ± 10.7	20.9 ± 3.3	p = 0.67
BMI (kg/m^2^ ± SD)	Total	28.6 ± 7.4	27. 9 ± 6.8	27.1 ± 5.6	p = 0.43
	T1D	26.9 ± 7.4	26.6 ± 7.4	25.6 ± 3.4	p = 0.75
	T2D	31.0 ± 7.7	29.5 ± 5.8	28.1 ± 6.7	p = 0.18
Established cardiovascular disease (%)	Total	35 (26.5)	43 (33.1)	26 (42.6)	p = 0.08
	T1D	13 (18.8)	13 (20)	8 (33.3)	p = 0.31
	T2D	21 (35)	30 (46.9)	18 (50)	p = 0.26
Antiplatelet use (%)	Total	48 (36.4)	49 (37.7)	28 (45.9)	p = 0.43
	T1D	17 (24.6)	20 (30.1)	9 (37.5)	p = 0.46
	T2D	88 (66.7)	29 (45.3)	19 (52.8)	p = 0.75
Lipid lowering therapy use (%)	Total	30 (50)	84 (64.6)	42 (68.9)	p = 0.84
	T1D	36 (52.2)	37 (56.9)	17 (70.8)	p = 0.28
	T2D	50 (83.3)	47 (73.4)	24 (66.7)	p = 0.16
Anti-hypertensive use (%)	Total	81 (61.4)	83 (63.8)	41 (67.2)	p = 0.73
	T1D	30 (43.4)	30 (46.2)	17 (70.8)	p = 0.06
	T2D	50 (83.3)	53 (82.8)	23 (63.9)	*p = 0.05*
Type 2 diabetes receiving insulin (%)		48 (80)	53 (82.8)	26 (72.2)	p = 0.45

Data are presented as number (%) or mean ± SD

Numbers in italics signify statistically significant differences

In the interventional part of the study, no statistical differences were observed in any variable when comparing the intervention and standard groups alone.

### Follow up period

Follow up periods (months) were 41.0 ± 16.2, 40.6 ± 16.2 and 50.2 ± 10.0 for interventional, standard and observational groups respectively.

### Mortality

In total, across all 3 groups, there were 90 deaths (27.9%) of whom 30 died in the first 12 months following ambulance call out (9.3%).

Comparing the intensive and combined arms, 28 (21.2%) and 62 (32.4%) deaths occurred respectively, during study period (p = 0.022). The risk ratio (RR) for all-cause mortality comparing intensive and combined arms was 0.65 (95% CI 0.44–0.96). A similar pattern was seen, albeit non–significant, comparing intensive to standard study arms of the interventional study, 28 (21.2%) and 39 (30.0%), respectively, p = 0.10) with RR for all-cause mortality of 0.71 (0.46–1.08).

When type 1 and type 2 diabetes individuals were separately analysed, a clear difference was demonstrated. In individuals with type 1 diabetes, mortality in the intensive arm at 11.6% was similar to those receiving standard management, regardless whether it was analysed as a combined group (mortality 12.4%) or when compared with the standard arm of the interventional study (mortality 10.8%). In individuals with type 2 diabetes, mortality in the intensive arm was lower at 33.3% than the combined group (51.2%; p = 0.025) with a similar trend observed when compared with the standard arm of the interventional study (50.0%; p = 0.06).

**Fig. 2:**
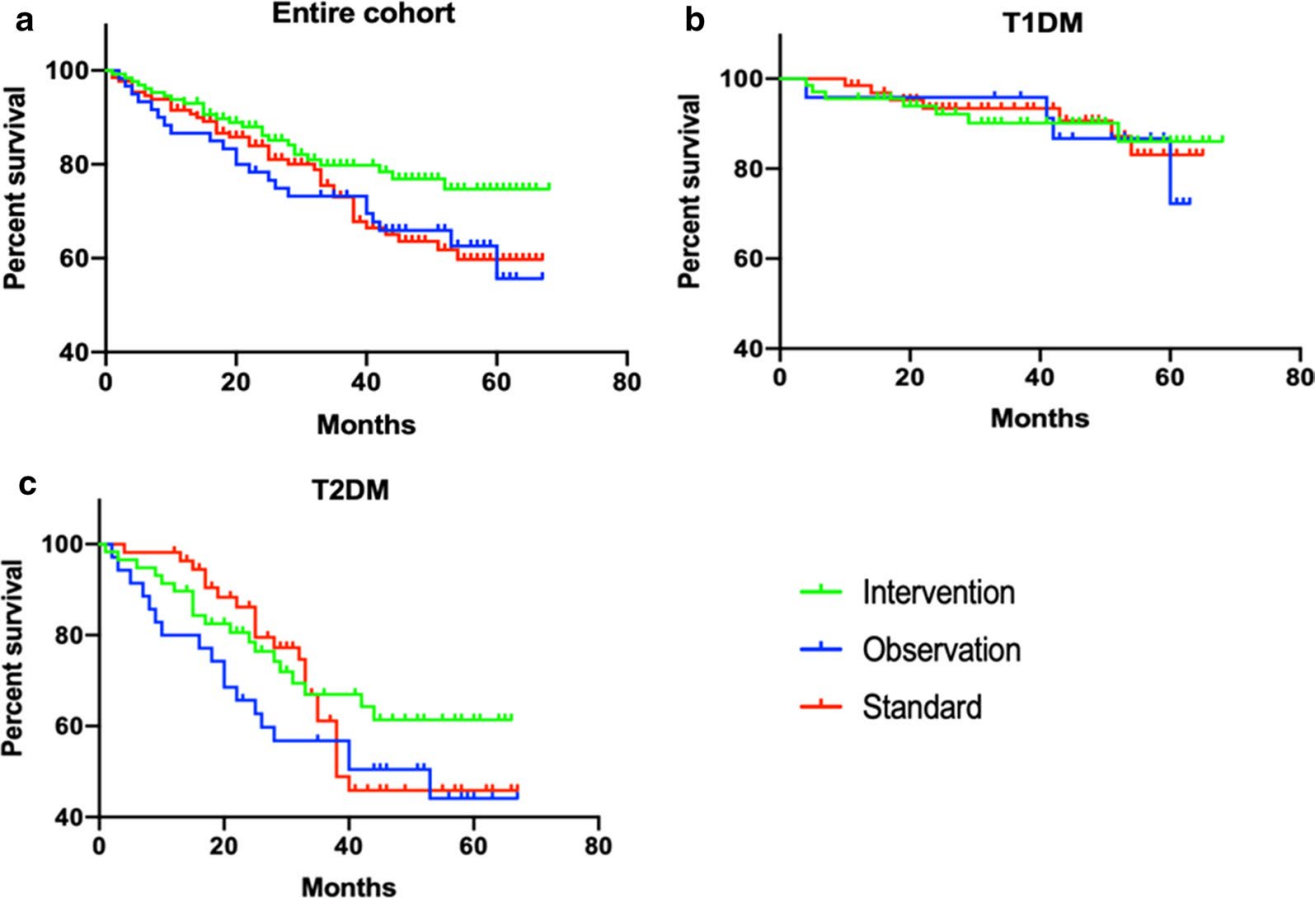
Kaplan Meier survival curves for all-cause mortality. Participants were followed up for a mean of 42.6 ± 15.6 months. **a** All individuals in the study. **b** Individuals with type 1 diabetes. **c** Individuals with type 2 diabetes. The green line denotes participants in the intensive arm of the study, the blue line those in the standard arm and the red line the observational arm

Kaplan Meier survival curves show mortality for all three groups when analysed as a whole group (Fig. 2a), for those with type 1 diabetes (Fig. 2b) and those with type 2 diabetes (Fig. 2c).

### Cause of death

The leading cause of death overall was cardiovascular disease (30% of cases) with rates of this outcome being significantly lower in the intensive arm of the interventional study (2.3% of participants) compared to standard and combined groups (10.8% and 13.1%, respectively p < 0.05).

### Cause of death in type 1 diabetes

Overall, mortality in participants with type 1 diabetes during study follow up was 12% and showed no difference between the groups (detailed above). Cause of death was similar across study groups with no trend identified. Of note, both of the deaths attributed directly to a glycemic emergency were seen in those with type 1 diabetes (one in intensive and one in standard study arms).

Mean age of death was 62.7 ± 14.9 years and mean time between enrolment to study and death was 26.2 ± 19.7 months.

Of note, those with type 1 diabetes in the study were younger and less likely to have established CV disease when compared to their counterparts with type 2 diabetes.

#### Cause of death in type 2 diabetes

Mortality in type 2 diabetes was much higher (44.3% across all groups) and in contrast to type 1 diabetes, showed differences between study arms. Cardiovascular disease was the leading cause of death in those with type 2 diabetes followed by infection. There was a significant reduction in death from a cardiovascular cause when comparing those in the intensive arm to those in the combined or standard groups (p < 0.01 for both). A separate analysis was conducted on those without clinically established CV disease to understand the role of intervention in this subgroup. A numerical decrease in mortality was evident in the interventional compared with the standard arm and while this failed to reach statistical significance, it was consistent with overall study findings (mortality at 28.2% and 41.0%, respectively; p = 0.24). Moreover, there was a statistically significant difference in rates of CV death comparing interventional and standard arms in this subgroup of individuals (0% vs. 14.7%, respectively; p = 0.01).

When further analysing the main cause of cardiovascular death in those with type 2 diabetes, we determined whether this was due to a cardiac, cerebrovascular or peripheral vascular cause. For the interventional group, the lone cardiovascular death was caused by coronary artery disease. For the standard group, 10 participants had a cardiac cause listed as the main cause of death, while in two participants cerebrovascular disease resulted in death. For the observational group, the main cause of death was cardiac in nine participants, cerebrovascular in one participant and peripheral vascular in one participant.

Mean age at death was 78.0 ± 8.1 years across all groups. Mean time between enrolment in the trial and death was 20.1 ± 13.6 months.

A summary of data for the cause of death in those with type 2 diabetes is shown in Table 2.

**Table 2:** A summary of cause of death in those with type 2 diabetes

Death from	Intensive (n = 60)	Standard (n = 64)	Observation (n = 36)	Test of difference: intensive vs. standard	Test of difference: intensive vs. combined
Cardiovascular cause (%)	1 (1.7)	12 (18.8)	11 (30.6)	*p = 0.002*	*p ≤ 0.001*
Infection (%)	7 (11.7)	9 (14.1)	3 (8.3)	p = 0.69	p = 0.95
Renal disease (%)	3 (5)	0 (0)	0 (0)	p = 0.11	p = 0.05
Old age/dementia/cancer (%)	4 (6.7)	6 (9.4)	2 (5.6)	p = 0.75	p = 1.0
Glycaemic emergency (%)	0 (0)	0 (0)	0 (0)	NA	NA
Other/unknown cause (%)	5 (8.3)	5 (7.8)	3 (8.3)	p = 1.0	p = 1.0
Total (%)	20 (33.3)	32 (50)	19 (52.8)	*p = 0.056*	*p = 0.025*

Cardiovascular death was the leading cause of fatality which was reduced with nurse led intervention in those with type 2 diabetes

Numbers in italics signify statistically significant differences

### HbA1c

Baseline HbA1c was available in 89% of all trial participants with a mean 62.7 ± 16.4 mmol/mol (7.9%). In those with type 2 diabetes, mean HbA1c was 59.7 ± 16.1 mmol/mol (7.6%), lower than those with type 1 diabetes 65.8 ± 16.4 mmol/mol (8.2%) (p < 0.01).

There was no difference in baseline HbA1c in those with type 2 diabetes in any of the trial arms. In those with type 2 diabetes, mean baseline HbA1c in those whom survived till study completion was 63.7 ± 16.4 mmol/mol (8.0%) compared with 59.8 ± 16.2 mmol/mol (7.6%) in those whom died at any point, a difference that failed to reach statistical significance (p = 0.079).

### Dropout rate

In the intensive arm of the study, a total of 7 participants failed to complete 3 months of the study, of whom 3 died, but all attended at least one study visit. No participant withdrew consent from any of the three study arms.

## Discussion

We and others have previously shown that mortality following severe hypoglycemia in the community is associated with high mortality, particularly cardiovascular death [25–27] but studies investigating modulation of this adverse clinical outcome are lacking. We present the first study that analyses the role of structured nurse-led intervention on mortality following severe hypoglycemia requiring ambulance services intervention. Over a follow up period of approximately 3.5 years, mortality occurred in 28% of study population, largely as a result of a high death rate in those with type 2 diabetes. In those whom received no intervention and had type 2 diabetes, half died within the study period, which dropped to a third following a simple nurse-led intervention. In contrast, nurse-led intervention had no effect on mortality in those with type 1 diabetes.

In the type 2 diabetes group, lowest mortality was observed in the intervention arm of the study, while mortality in the standard and observational arms was very similar. Importantly, a reduction in cardiovascular mortality was demonstrated between the standard and intervention study arms whom were well-matched at baseline and had an almost identical duration of follow up. Hypoglycemia is particularly detrimental in those with type 2 diabetes and clinically established cardiovascular disease [28]. However, our analysis suggests that cardiovascular mortality is reduced in the intervention arm regardless of the presence of clinically apparent vascular disease at baseline, which may indicate that vascular pathology was underdiagnosed in our cohort or alternatively the benefits are evident even in those with subclinical disease. Of note, type 2 diabetes individuals in the observational arm had the highest mortality, which may have been due to the older age but numbers are relatively small to draw definitive conclusions.

These data emphasise the high death rate in individuals with type 2 diabetes and severe hypoglycemia but also show that this is potentially modifiable using a relatively simple intervention and regular nurse follow up. It should be stressed that individuals in the standard and observational arms received support from their healthcare professional according to established guidelines. This included a minimum of one patient contact with subsequent tailored management according to the need of each individual. Therefore, the difference may have been even larger in areas where no contact is made when patients suffer severe hypoglycemia. The main differences between intensive and standard study arms were related to: (i) a systematic approach for establishing the cause of hypoglycemia and provision of simple education, (ii) encouragement to undertake regular glucose testing, and (iii) frequent nurse contact for 3 months following the hypoglycemic event. The failure to show a survival benefit for the nurse-led intervention in individuals with type 1 diabetes may be related to the limited number of events in this group and lack of adequate power to demonstrate a difference. Alternatively, it may be due to better hypoglycemic knowledge in this population with the nurse support having little additional benefit. It is also possible that the type 1 diabetes group had less extensive vascular disease, which limited the detrimental effect of hypoglycemia.

A striking observation is the continued drop in mortality beyond the period of nurse intervention, suggesting that a short period of structured nurse support is enough to reduce long-term mortality in the type 2 diabetes group.

HbA1c was lower at baseline in individuals with type 2 diabetes compared to those with type 1 diabetes, indicating that hypoglycemia requiring ambulance services intervention occurs with higher average glucose control in those with type 1 diabetes. Moreover, there was a numerical inverse relationship between baseline HbA1c and mortality in type 2 diabetes suggesting that tight glycaemic control in the older population is best avoided.

When investigating the cause of death in type 2 diabetes individuals, cardiovascular disease was the commonest cause followed by infections. Post-hoc analysis of the Veterans Affairs Diabetes Trial [29] also found increased risk of cardiovascular events in those whom had suffered an episode of severe hypoglycemia in the previous 3 months and these findings are consistent with reports from other large-scale trials [12]. Interestingly, reduction in mortality was mainly due to lower cardiovascular events in the intensive nurse-led arm of the study, while having no effects on other causes of death. Of note, the main role of the nurse was to focus on optimising glycaemic control, while avoiding interference with the management of other vascular risk factors. Therefore, the difference in mortality comparing study arms is likely related to glycemic factors, although patient-related behavioural changes (such as decreased alcohol consumption) may have also played a role.

The work has a number of strengths to highlight. First, no interventional study to date has investigated modulation of outcome in individuals with severe hypoglycemia requiring emergency services intervention. Second, individuals were well characterised, including classification into type 1 diabetes and type 2 diabetes, while the cause of death was clearly established. Third, median study follow up extended to over 3 years with an effect for the intervention shown well beyond the period of intensive nurse support. Fourth, retention in the study was high, indicating that the intervention is acceptable and convenient to patients, and therefore can be rolled out to the wider population.

There are a number of limitations to the study that should be acknowledged. First, more than half the patients approached declined to participate in the study or failed to attend study appointment, and therefore a significant proportion of patients may not wish to undergo such a nurse-led intervention. Second, this was a pilot study covering one geographical area and the number of individuals in each study arm was relatively small. In particular, mortality in individuals with type 1 diabetes was low and therefore no concrete conclusions can be drawn in this group. Third, the main focus of the intervention was to provide education to participants and encourage regular glucose testing; therefore the exact details of changes to patient medications, including insulin, were not systematically recorded. As a result, we are unable to report on changes in total daily insulin doses or alterations to oral hypoglycemic agents or indeed the effect of the intervention on reducing further hypoglycemic episodes. This will require assessment in future large scale trials, perhaps with the aid of continuous glucose monitoring to accurately study hypoglycemic exposure in study arms. Fourth, unlike the interventional and standard study arms that were well-matched, there were some differences in baseline patient characteristics in the observational arm. Therefore, we should be cautious in our interpretation when comparing interventional with the combined standard and observational groups. However, the interventional arm consistently showed lower mortality, whether compared with the combined group or the standard group, strongly suggesting a beneficial effect of the intervention on survival. Fifth, we do not provide mechanistic explanations for the apparent survival benefit of the intervention, although modulation of hypoglycemia and/or glycemic variability may have contributed to the beneficial vascular effect [30] but data on these glycemic variables were not collected. Finally, it was not possible to blind participants to study arms, given the nature of the work, and therefore the role of altered patient behaviour to study outcome remains unclear.

In summary, this pilot study raises the exciting possibility that short-term intensive nurse led intervention can reduce cardiovascular mortality in a high-risk group of individuals with type 2 diabetes following an episode of hypoglycemia requiring ambulance call out. Further large-scale trials, focussed on individuals with type 2 diabetes, are warranted to confirm results and assess the cost effectiveness of such an approach. Also, future studies should collect data on changes to hypoglycemic medications, including alterations to insulin type or dose, as well as substitution of oral treatment with agents that have low risk of hypoglycemia. This is particularly relevant given recent data showing that sodium-glucose transport protein-2 inhibitors and glucagon-like peptide-1 analogues are associated with favourable cardiovascular outcome, which may further contribute to reduction in vascular events, the main cause of mortality in this population.

## Conclusions and further work

This pilot study extends previous findings that severe hypoglycemia in the community represents significant danger to those with type 2 diabetes with a particularly concerning high rate of cardiovascular death over a short to medium follow up period. This work suggests that this increased risk is potentially modifiable with intensive nurse led intervention and further larger trials are required to investigate generalisability of our findings to different healthcare models.

## Data Availability

The datasets used and/or analysed during the current study are available from the corresponding author on reasonable request.
